# Commentary: A novel signature of 23 immunity related gene pairs is prognostic of cutaneous melanoma

**DOI:** 10.3389/fimmu.2023.913339

**Published:** 2023-06-30

**Authors:** Ruihuan Shen, Tong Zou

**Affiliations:** Department of Cardiology, Beijing Hospital, National Center of Gerontology, Institute of Geriatric Medicine, Chinese Academy of Medical Sciences & Peking Union Medical College, Beijing, China

**Keywords:** signature, immunity, gene pairs, prognostic, cutaneous melanoma

## 1 Introduction

This paper is a commentary on the article “A Novel Signature of 23 Immunity Related Gene Pairs Is Prognostic of Cutaneous Melanoma” (Ya-Nan Xue et al., 2020) (1). The authors aimed to identify an immune-related signature for predicting prognosis in cutaneous melanoma (CM). But there are some statistical errors in the statistical inference process, which mainly reflect in the correlation between interval estimation and hypothesis testing showing in TABLE 2 presenting Univariate Cox and Multivariate Cox analysis of clinicopathological factors and risk signatures. Through the following discussion of this published paper, we can be more familiar with the correlation between interval estimation and hypothesis testing, and avoid errors in statistics.

## 2 Statistical errors in the published paper

It’s clear that there are some statistical errors in TABLE 2. For example, in the TCGA dataset, the 95% confidence intervals (CI) of the hazard ratios (HRs) of age and stage in Multivariate Cox analysis of clinicopathological factors and risk signatures are 0.98 - 1.04 and 0.83 - 1.43, respectively, but p-value = 0.010 and 0.008, respectively; Moreover, in the GSE59455 validation dataset the 95% CI of the HR of risk Score in Multivariate Cox analysis of clinicopathological factors and risk signatures is 0.94 - 2.34, but p-value = 0.042. It is known that the range of this 95% CI includes 1, so this test should not be statistically significant (p-value > 0.05). Then, our task group had attempted to reproduce the statistical analyses. However, the results demonstrate the validity of our original vision that there are some numerical results in the original paper and the 95% CI for HR which includes HR = 1, certainly couldn’t has a corresponding p-value< 0.05. (the data have not yet been published). Next, to confirm the validity of our conclusions, we explain the concepts of interval estimation and hypothesis testing, as well as the relationship between them in detail later in this commentary.

## 3 The concept of interval estimation

Interval estimation involves an interval that measures the accuracy of a point estimate. The point estimate of parameter is calculated to get a specific value, which is easy to calculate and use. However, the accuracy of the calculated point estimation cannot be explained by the point estimation itself, and it needs to be reflected by the corresponding distribution. In reality, we usually use an interval to cover the known point estimation, and we can also say that we can get the range of the value of the point estimation, it should be a range, as defined below:

Let *θ* be a parameter of the population, x_1_, x _2_,…, x_n_ be the sample, obtain the statistics *θL* (x_1_, x_2_,…, x_n_), *θU* (x_1_, x_2_,…, x_n_), make *θL* < *θU*; get the sample observations, then *θ* is estimated in the interval [*θL*, *θU*]. Because of the randomness of the sample, the probability of the cover parameter *θ* is uncertain, and we usually need to give an artificial probability here. Of course, we certainly want the probability *P* of the cover *θ* to be as large as possible, but this will inevitably lead to an increase in the length of the interval, where the probability of the interval [*θL*, *θU]* covering θ is given to 1-α, then P _θ_ (*θL* ≤ *θ* ≤ *θU*)≥1-α, then the confidence level of the random interval [*θL*, *θU*] is called the confidence level of *θ* of 1-α, *θL* and *θU* are called *θ*’s (bilateral) lower and upper confidence limits, respectively.

## 4 The concept of hypothesis testing

The hypothesis testing is a process of testing the validity of hypotheses. It is a statistical inference method used to judge whether the differences between samples and population are caused by sampling errors or by the essential differences between samples. There are many methods of hypothesis testing. As one of the most commonly used hypothesis testing methods, the basic principle of significance test is to make some assumptions about a parameter of the population based on some non-sample information, and then through the statistical reasoning of sampling information, make a judgment on whether this hypothesis should be rejected. For example, gene sets with an FDR-adjusted probability (p) value< 0.05 were considered statistically significant in this published article. That said, when p value was greater than 0.05, the null hypothesis should be rejected.

## 5 The relationship between interval estimation and hypothesis testing

It is worth mentioning that there is a very close relationship between the CI of normal population parameters and the test of hypothesis testing. The similarity between the pivotal quantity and test statistics is not accidental, but there is a one-to-one correspondence between the two statistical inference methods (2). The two problems can be transformed into each other. The derivation is as follows:

In Two-side test:

Null hypothesis (H_0_): *μ* = *μ*_0_ Alternative hypothesis(H_1_): *μ* ≠ *μ*_0_

The test statistic: $u=\sqrt{n}\frac{\bar{x}-\mu_{0}}{\sigma }$ ; Rejection region *W*: $\left\{\left|u\right|≽u_{1-\frac{\alpha }{2}}\right\}$ ; Acceptance region $\bar{W}:\left\{\left|u\right|≼u_{1-\frac{a}{2}}\right\}$ , that is, $\left\{\left|\left(\sqrt{n}\frac{\bar{x}-\mu_{0}}{\sigma }\right)\right|≼u_{1-\frac{\alpha }{2}}\right\}$ , It can eventually be rewritten as $\left\{\bar{x}-\frac{\sigma \times u_{1-\frac{\alpha }{2}}}{\sqrt{n}}≼\mu_{0}≼\bar{x}+\frac{\sigma \times u_{1-\frac{\alpha }{2}}}{\sqrt{n}}\right\}$ , which is the 1-α CI for the population parameter μ.

Consequently, Under the same condition, the 1-α CI problem in interval estimation corresponds to the hypothesis test problem with the significance level α, and the result is the same. Therefore, the range of this 95% CI includes 1, which has been equated with *P* > 0.05, so this test should not be statistically significant.

## 6 Discussion

The p value and CI are the two most widely used statistical inference tools. The American Statistical Association issued a warning statement on the use of the p value due to the misuse and abuse of the p value (3). There are some mistakes in understanding the p value and CI which caused the wrong use of the p value (4). Furthermore, the researchers may have never considered the relationship between the p value and CI. Therefore, this paper expects to introduce the definition of p value and CI through the analysis of that previously published paper mentioned above, and we also hope to analyze the relationship and difference between the p value and CI, to help researchers understand the p value and CI correctly.

## 7 Conclusion

Undoubtedly, the authors of that previously published article have failed in understanding the relationship between CI and p value. Only when we understand them correctly can we accurately use them to explain the research results and significance of the article, which is crucial to the quality of the article.

## Author contributions

All authors listed have made a substantial, direct, and intellectual contribution to the work and approved it for publication.
